# FMSM: a novel computational model for predicting potential miRNA biomarkers for various human diseases

**DOI:** 10.1186/s12918-018-0664-9

**Published:** 2018-12-31

**Authors:** Yiwen Sun, Zexuan Zhu, Zhu-Hong You, Zijie Zeng, Zhi-An Huang, Yu-An Huang

**Affiliations:** 1 School of Medicine, Shenzhen University, Shenzhen, 518060 China; 2 College of Computer Science and Software Engineering, Shenzhen University, Shenzhen, 518060 China; 3 Xinjiang Technical Institute of Physics and Chemistry, Chinese Academy of Science, ürümqi, 830011 China; 4 Department of Computer Science, City University of Hong Kong, Hong Kong, 999077 China; 5 Department of Computing, Hong Kong Polytechnic University, Hong Kong, 999077 China

**Keywords:** Biomarker, Computational prediction, miRNA-disease association, Expression profiles

## Abstract

**Background:**

MicroRNA (miRNA) plays a key role in regulation mechanism of human biological processes, including the development of disease and disorder. It is necessary to identify potential miRNA biomarkers for various human diseases. Computational prediction model is expected to accelerate the process of identification.

**Results:**

Considering the limitations of previously proposed models, we present a novel computational model called FMSM. It infers latent miRNA biomarkers involved in the mechanism of various diseases based on the known miRNA-disease association network, miRNA expression similarity, disease semantic similarity and Gaussian interaction profile kernel similarity. FMSM achieves reliable prediction performance in 5-fold and leave-one-out cross validations with area under ROC curve (AUC) values of 0.9629+/− 0.0127 and 0.9433, respectively, which outperforms the state-of-the-art competitors and classical algorithms. In addition, 19 of top 25 predicted miRNAs have been validated to have associations with Colonic Neoplasms in case study.

**Conclusions:**

A factored miRNA similarity based model and miRNA expression similarity substantially contribute to the well-performing prediction. The list of the predicted most latent miRNA biomarkers of various human diseases is publicized. It is anticipated that FMSM could serve as a useful tool guiding the future experimental validation for those promising miRNA biomarker candidates.

**Electronic supplementary material:**

The online version of this article (10.1186/s12918-018-0664-9) contains supplementary material, which is available to authorized users.

## Background

Over the last decade, huge progress has been achieved in understanding of a class of small (about 22 nucleotide), single-stranded non-coding RNAs, known as microRNAs (miRNAs) [1]. Since two members of the miRNA family (i.e., the products of the *Caenorhabditis elegans* genes lin-4 and let-7) were firstly identified in [2–4], over 2000 miRNA sequences have been reported in human genome [5]. miRNAs primarily get involved in the negative regulation of gene expression. Their mediated regulation plays a key role in a wide range of biological processes, such as metabolism, apoptosis, developmental timing, neuronal gene expression, stem cell maintenance, host-viral interaction, cardiac and skeletal muscle proliferation [6, 7]. Increasing studies suggest much diverse mechanisms of miRNA action, including binding to the 5’UTR of ribosomal protein mRNAs and coding region with functional consequences [8]. It is estimated that about 50% protein coding genes are regulated by miRNAs in mammals [7, 9–11]. It is realized that the characterization of miRNAs is much more important than previously thought in gene expression regulation, the evolution of species, the origin of life and, disease mechanisms and development [10].

Further studies uncover not only their roles in diverse cellular processes, but also the abnormal patterns of miRNA expression in various human clinical diseases, such as inherited diseases (e.g. hereditary progressive hearing loss [12] and skeletal and growth defects [13]), heart disease [14], kidney disease [15], obesity [16], alcoholism [17], nervous system (e.g. Alzheimer disease [18] and schizophrenia [19]) and cancer (e.g. chronic lymphocytic leukemia [20] and colorectal cancer [21]). For example, a number of miRNAs have been regarded as “tumor suppressive miRNAs” or “oncomiRs” [22]. In malignant B cells, some miRNAs (such as miR-150, miR-155, miR-21, miR-34a, miR-17-92 and miR-15-16) are involved in pathways fundamental to B-cell development like B-cell migration/adhesion, the production and class-switching of immunoglobulins, B-cell receptor (BCR) signaling, and cell–cell interactions in immune niches [20]. By analyzing the miRNA expression levels and the corresponding patients’ survival, these “oncomiRs” are anticipated to be used as predictive and prognostic markers. In 2009, a study on inhibiting the metastatic nature of breast cancer suggested that five members of the microRNA-200 family are down-regulated in tumor development of breast cancer [23]. These convincing evidences prove that miRNAs could serve as master regulators of gene expression in multiple disease-related signaling pathways. Specifically, miRNA signatures or expression levels are emerging as promising biomarkers for disease therapy, diagnosis, prognosis and prevention.

However, the mechanisms among the miRNA-disease associations remain unclear. The traditional biological experiments are costly, laborious and time-consuming. There is a great need to develop an effective and efficient way to facilitate the identification of latent disease-related miRNAs. With the advances of high-through sequencing technology [24] and bioinformatics, researchers shift the focus on the relationships between miRNA dysregulation and human diseases from different perspective. Dozens of publicly available databases or webservers have been set up to archive diverse types of biological information. For examples, miRBase [5] is the primary repository providing miRNA sequence and annotation data. miRTarBase [25] has accumulated more than 3500 miRNA-target interactions (MTIs). starBase [26] was developed to comprehensively explore miRNA-target interaction maps from CLIP-Seq and Degradome-Seq data. MicroRNA.org [7] incorporates miRNA target predictions and expression profiles. miR2Disease, dbDEMC and HMDD are manually curated databases collecting experimentally verified miRNA-disease associations with corresponding literature references [27–29].

The publicly available databases are essential to provide opportunity for developing computational models of large-scale related relation inference. It inspires researchers to preferentially conduct research on the biological interpretation of high-scoring candidate inferred by the computational prediction [30–32]. In recent years, a number of computational models have been presented to predict the most possible disease-related miRNAs. Based on the miRNA similarity derived from various data sources, these models could be classified into three main categories. The first category is mainly based on the miRNA functional similarity. For example, Jiang et al. [33] leveraged a functionally related network to measure functional relatedness between any two investigated miRNAs. Based on the hypothesis that functionally related miRNAs tend to have a close relationship with phenotypically similar diseases, the potential miRNA-disease associations can be prioritized by integrating the phenome-miRNAome network. However, the performance of Jiang’s model is limited because the predicted miRNA-target associations they utilized inevitably include a high rate of false-positive and false-negative samples. The second category was developed for protein-driven inference. Mørk et al. [34] presented a computational model of miRNA-Protein-Disease associations called miRPD by coupling protein-disease text mined from the literature with known or predicted miRNA-protein associations. They also devised a scoring schemes to rank potential miRNA-disease associations based on the reliability, so high- and medium-confidence sets of associations could be created. The third category was developed by introducing multiple data sources, such as miRNA-lncRNA associations, miRNA target-dysregulated network (MTDN), miRNA and mRNA expression profiles. Liu et al. [35] established the miRNA similarity network composed of the miRNA-target gene, miRNA-lncRNA associations and lncRNA-disease associations. Then they extended random walk with restart to infer miRNA-disease associations in the heterogeneous network. Shi et al. [36] also used random walk analysis to measure the potential regulatory relationship between miRNA and disease by exploiting the functional relatedness between disease genes and miRNA targets in protein-protein interaction (PPI) network.

To the best of our knowledge, no existing computational model has been presented considering the similarity of expression distribution of diverse miRNAs in human tissues. Moreover, most of the previous computational models were devised to prioritize the most latent miRNA-disease associations among all unknown pairs and thereby adopt the global scoring schemes, which could not be suitable for top-N recommendation for each disease. Actually, this research topic could be considered as matrix filling problem, for which most algorithms in recommender system work well. Kabbur et al. [37] proposed an item-based model called FISM allowing two matrices to learn the item similarities. The product of these two matrices was used for yielding top-*N* recommendations. The effectiveness of this model was demonstrated, especially for sparse datasets. Based on this work, we present a novel computational model named FMSM for predicting potential miRNA biomarkers of various human diseases, rather than the miRNA-disease candidate associations for all considered diseases. FMSM is proposed to extend our previous work (PBMDA [38]). Since the target is different from the previous work, using local scoring scheme is more suitable. FMSM is a Factored MiRNA Similarity based Model. Based on the known miRNA-disease associations, FMSM learns the miRNA similarities as the product of two latent factor matrices for certain disease using a structural equation modeling approach. By integrating miRNA expression similarity, disease semantic similarity and Gaussian interaction profile kernel similarity, the experimental performance suggests that the proposed model could manage sparse datasets effectively. It also has been proved by the experiment result that PBMDA performs worse in local LOOCV although works well in global LOOCV. Since the local scoring scheme adopted in the proposed model, FMSM obtained significant improvement over PBMDA and other state-of-the-art computational models. Based on two validation frameworks of leave-one-out cross validation (LOOCV) and 5-fold cross validation (5-fold CV), FMSM obtained the highest AUC values of 0.9433 and 0.9629+/− 0.0127 respectively. To further assess the performance of FMSM, we also implemented a case study of an important human disease. Moreover, the novel feature miRNA expression similarity was introduced in this model and was demonstrated to have better capability of characterizing the miRNA function and nature via the contrast experiment. We have publicly released the list of the most latent miRNA biomarkers predicted for various human diseases (see Additional File 1), which is expected to provide an insight into the miRNA therapeutic modulation as anti-disease agents with further experimental validation.

## Results

### Leave-one-out and 5-fold cross validation

Two validation frameworks, i.e., LOOCV and 5-fold CV were employed to assess the predictive performance of the proposed model based on the known miRNA-disease associations derived from HMDD v2.0 database [29]. Since the proposed model aims to predict the potential miRNA biomarkers for various human diseases, the predictive score of the test sample is only compared with other candidate miRNAs’ in the scope of the same disease. This type of LOOCV is so called local LOOCV. In the framework of local LOOCV, each known miRNA-disease association is used as a test sample in turns while other known miRNA-disease associations are used to train the model. In the framework of 5-fold CV, we randomly divided all known miRNA-disease associations into five uncrossed groups. Similarly, each group serves as the test samples and the other four groups serve as the training samples. To reduce bias brought by sample divisions, we repeated experiments of 5-fold CV for 20 times and that the average value was calculated as the final evaluation index representing the performance of 5-fold CV. If the score of the test sample is ranked higher than a specific parameter, the proposed model makes a successful prediction.

The receiver operating characteristic (ROC) curve and AUC are commonly used to evaluate the predictive performance of binary classification problems. ROC curve and AUC can be used to directly observe the experiment results by visual picture and numerical value, respectively. ROC curve can be drawn by simultaneously computing the true positive rate (TPR, sensitivity) and false positive rate (FPR, 1-specificity) according to the varying parameter. Sensitivity and specificity are statistical measures formulated as follows:

1 $$ {\displaystyle \begin{array}{l} SEN=\frac{TP}{TP+ FN}\\ {} SPE=\frac{TN}{TN+ FP}\end{array}} $$

where *TP*, *TN*, *FP* and *FN* are abbreviations of the number of true positive, true negative, false positive and false negative respectively. In this way, the ROC curve can be plotted parametrically based on TPR versus FPR. Generally, AUC = 1 indicates a perfect prediction while AUC = 0.5 indicates an entirely random one.

**Fig. 1:**
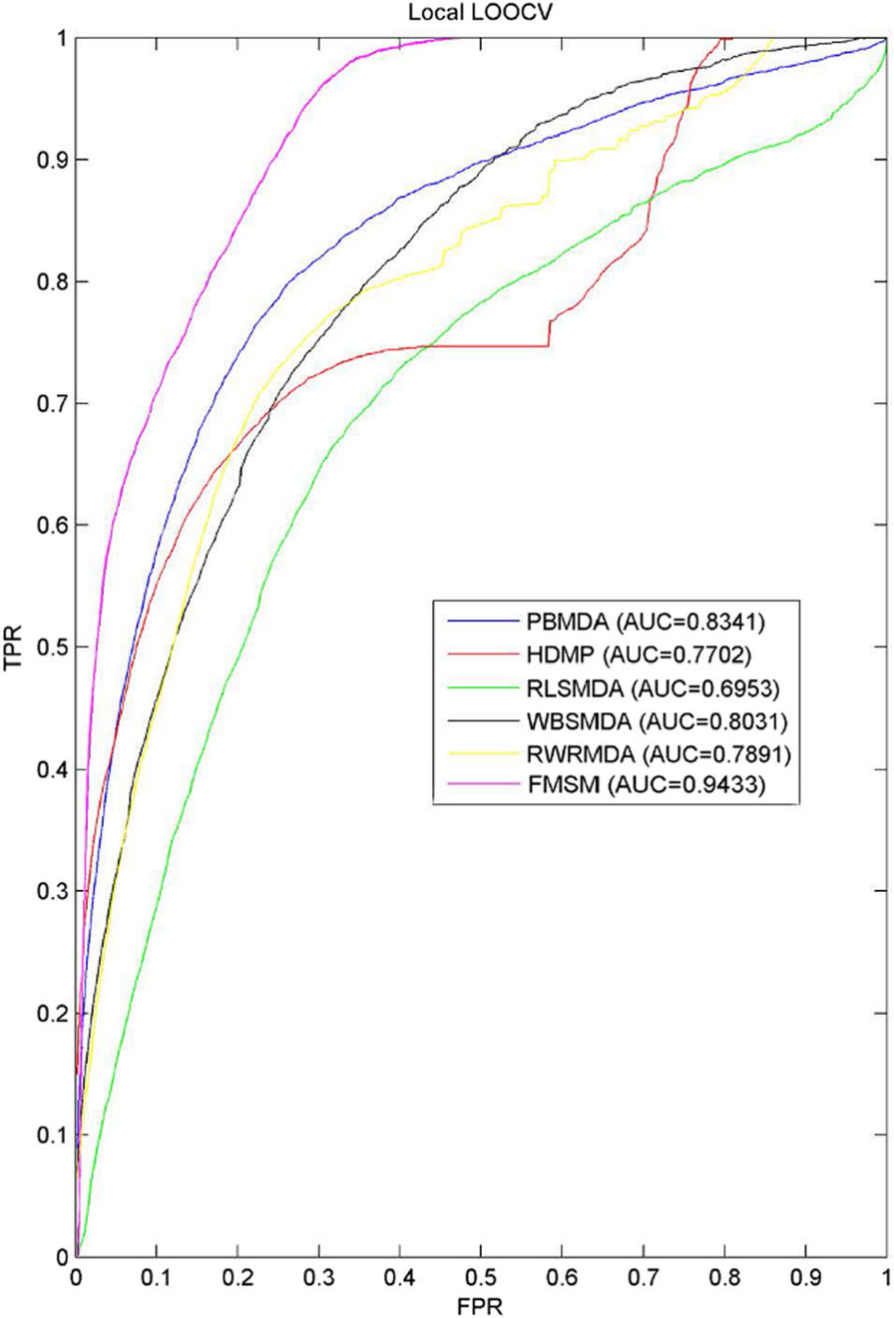
The comparison results between FMSM and other five state-of-the-art computational models in the framework of LOOCV

A few state-of-the-art computational models [38–42] have been proposed for miRNA-disease association prediction based on HMDD v2.0, which is the same information source of FMSM. Based on the hypothesis that miRNAs with similar functions often have close associations with similar diseases, all of these tested models inferred the pairwise miRNA functional similarity by Wang’s method [43]. To evaluate the performance of FMSM, five state-of-the-art models namely PBMDA [38], HDMP [42], RLSMDA [39], WBSMDA [40], and RWRMDA [41] were also tested and compared with FMSM via local LOOCV (see Fig. 1). The results of FMSM and all state-of-the art compared models were tested on the same evaluation program in LOOCV for ensuring the fair comparison. HDMP and RWRMDA are both representational models in this domain. HDMP uses the information of the most weighted similar neighbors for inference. RLSMDA can be regarded as a good trial in machine learning algorithm using Regularized Least Squares (RLS). By fusing heterogeneous biological information, WBSMDA leverages an efficient formulation of calculating and combing within-score and between-score for the prediction. PBMDA represents the current level in this domain and adopts an effective path-based approach using a special depth-first search algorithm. It means that test samples were only ranked among other candidate miRNA-disease associations for a given disease, rather than all investigated diseases. As a result, PBMDA, HDMP, RLSMDA, WBSMDA, RWRMDA and FMSM achieved AUC values of 0.8341, 0.7702, 0.6953, 0.8031, 0.7891 and 0.9433 respectively. In a word, FMSM obtained the best prediction performance with the highest AUC of 0.9433 in local LOOCV, which demonstrated the reliable prediction of FMSM. The other compared methods were all used for prioritizing the most likely miRNA-disease associations based on the global measure-based scoring scheme, which could weaken the power of disease-specific prediction because of the disproportional coverage in known miRNA-disease association network. Moreover, the miRNA expression similarity we first introduced into FMSM could better characterize miRNA function and nature. We also implemented 5-fold CV on FMSM resulting in an average AUC value of 0.9629 with standard deviation of 0.0121. Since the competitors adopt global scoring schemes, their 5-fold CV prediction performance in terms of average AUC value was not provided in the literatures. Therefore, we could not compare FMSM with the competitors via 5-fold CV.

**Fig. 2:**
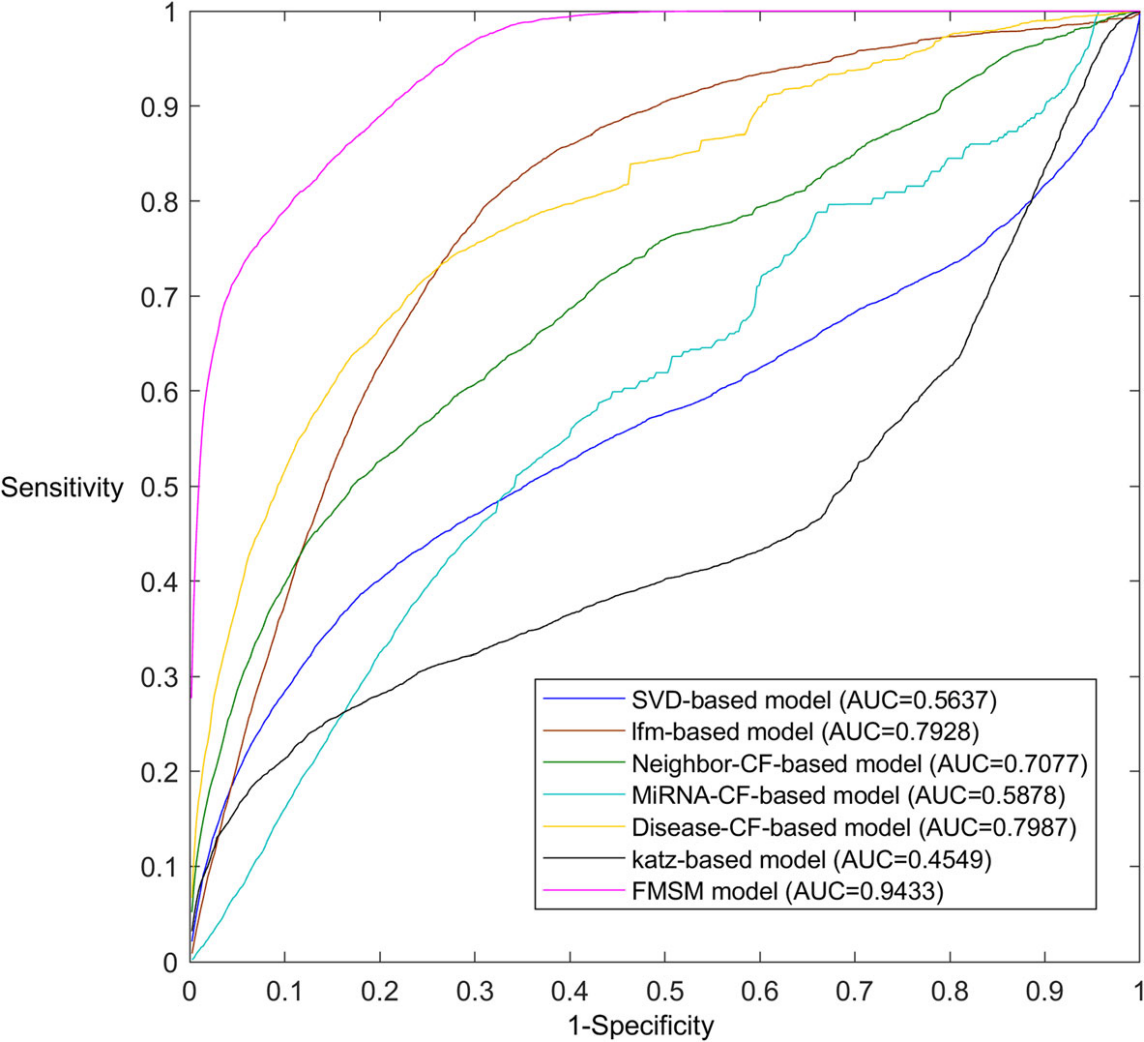
The comparison results between FMSM and other six classical algorithms in terms of LOOCV

Since miRNA-disease association prediction could be considered as a matrix filling problem, which is similar to recommendation system and social network recommendation. Some classical user-item based recommended algorithms (including svd-based model [44], latent factor model [45], neighbor-based collaborative filtering, user-based collaborative filtering and item-based collaborative filtering [46]) and social network prediction method (i.e., Katz-based model [47]) were also involved in the comparison with FMSM via local LOOCV (see Fig. 2). To apply user-item based recommended algorithms and social network prediction method, the solution should be converted into recommending the most potential miRNAs to certain diseases, like recommending favorite items to certain users in recommendation system and potential friends to certain users in social network. The fairness of the comparative experiments was ensured by using the same information source, i.e. the known miRNA-disease associations, miRNA expression similarity and disease semantic similarity. As we can see in Fig. 2, FMSM obviously outperforms the competitors achieving the highest AUC value 0.9433. The experimental result proves that other competing approaches fail to handle such sparse dataset and therefore generate low quality predictions. Moreover, they usually are used to make a faster recommendation but sacrificing accuracy to a certain extent. In conclusion, the reliable prediction performance shown in local LOOCV and 5-fold CV suggests that FMSM indeed improves the prediction accuracy compared with other state-of-the-art computational models.

### Case study

As we have mentioned before, a few miRNAs work as regulatory molecules in cancer, acting as tumor suppressors. Based on HMDD database, we implemented a case study of Colonic Neoplasms (CN) using the proposed model to explore the potential relationship between miRNA and the mechanisms of digestive cancer. The prediction list of CN in top 25 was validated via the other two independent databases (i.e., dbDEMC [28] and miR2Disease [27]). It needs to note that, all predicted miRNA-disease associations are excluded from HMDD database.

CN is the abnormal growth of cells that has the ability to invade to other parts of human body from colon or rectum [48]. Signs and symptoms could include feeling tired all the time, blood in the stool and weight loss. CN is the second leading cause of cancer death in the United States with five year survival rates of around 65% [49]. Vogelstein et al. [50] described that epigenetic alterations are much more frequent in CN than genetic (mutational) alterations and miRNA expression can be epigenetically altered. For example, silencing of miR-137 has been demonstrated to affect expression of about 500 genes, which may cause an early epigenetic alteration in CN [51]. Therefore, some miRNAs could be used as biomarkers applicable to the early diagnosis and prevention. As we can see in Table 1, 6 out of the top 10 and 19 out of top 25 predicted miRNAs are verified by dbDEMC and miR2Disease. It is anticipated that those unconfirmed miRNAs, especially which ranked in the 1st, 2nd, 4th and 6th, have a high probability to have a close relationship with CN and thereby deserve to be validated by further biological experiments.

**Table 1:** FMSM was applied to Colonic Neoplasms to prioritize the latent disease-related miRNAs. Six of top 10 and 19 of top 25 predicted miRNAs have been validated via dbDEMC and miR2Disease

Top 1–25					
Rank	miRNA	Evidence	Rank	miRNA	Evidence
1	hsa-mir-1909	unconfirmed	14	hsa-mir-182	dbDEMC;miR2Disease
2	hsa-mir-1183	unconfirmed	15	hsa-mir-27a	miR2Disease
3	hsa-mir-196a	dbDEMC;miR2Disease	16	hsa-mir-34c	miR2Disease
4	hsa-mir-1273c	unconfirmed	17	hsa-mir-30b	dbDEMC
5	hsa-mir-133a	dbDEMC;miR2Disease	18	hsa-mir-567	unconfirmed
6	hsa-mir-1179	unconfirmed	19	hsa-mir-34b	dbDEMC;miR2Disease
7	hsa-mir-206	dbDEMC	20	hsa-mir-15b	miR2Disease
8	hsa-mir-148a	dbDEMC	21	hsa-mir-124	dbDEMC
9	hsa-mir-218	dbDEMC	22	hsa-mir-1275	unconfirmed
10	hsa-mir-26a	dbDEMC;miR2Disease	23	hsa-mir-222	dbDEMC
11	hsa-mir-212	dbDEMC	24	hsa-mir-181b	dbDEMC;miR2Disease
12	hsa-mir-23a	miR2Disease	25	hsa-mir-429	dbDEMC
13	hsa-mir-210	dbDEMC			

### The effect of combining different miRNA similarities

**Fig. 3:**
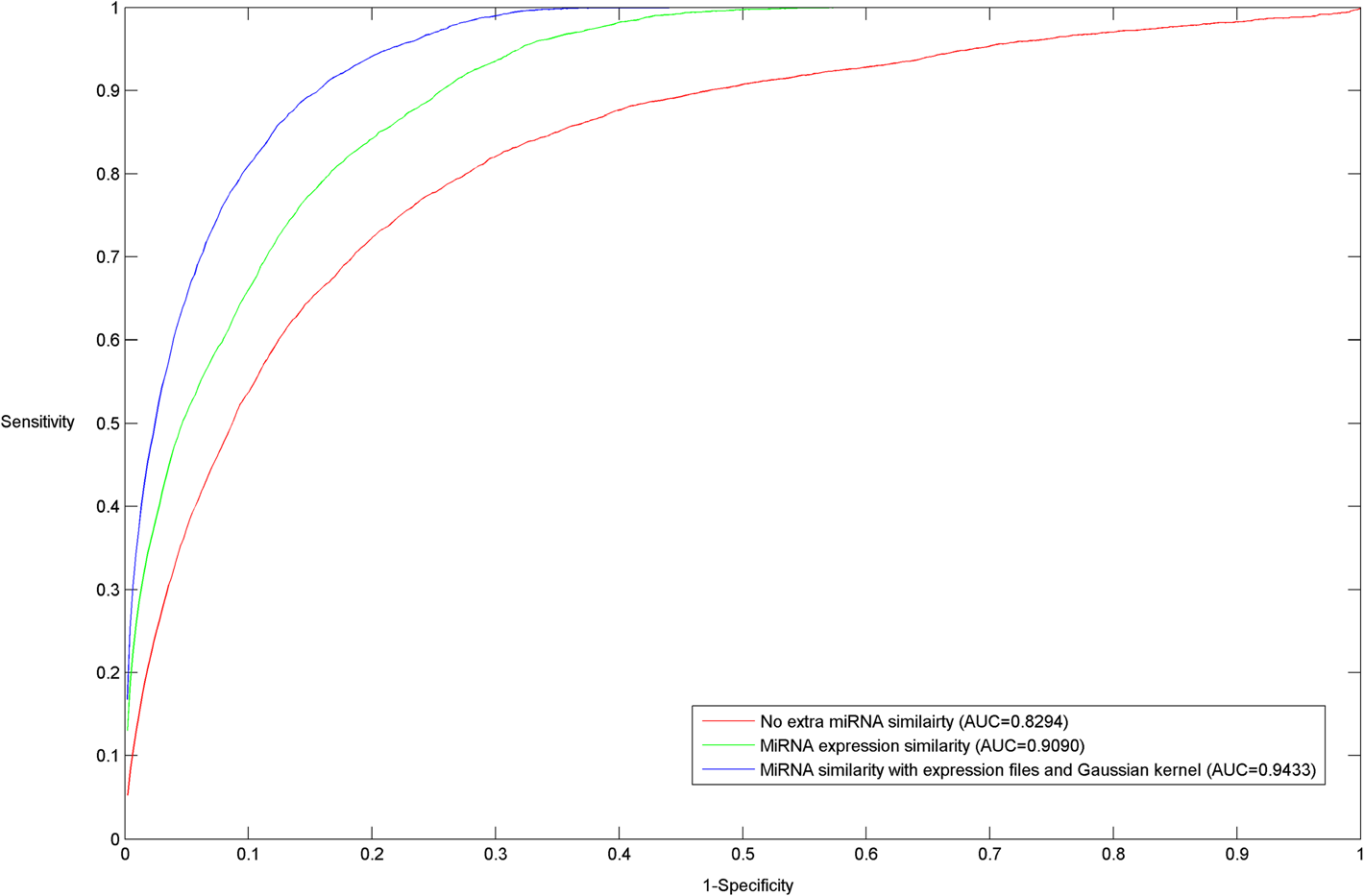
The effect of combining different miRNA similarities was tested via LOOCV

In this section, both local LOOCV and 5-fold CV were used to assess the effect of combining diverse types of miRNA similarities, i.e., no extra miRNA similarity, miRNA expression similarity, and miRNA similarity with expression files and Gaussian kernel (see Fig. 3 and Table 2). Except for the different input of miRNA similarity, other information source inputs were kept consistent, i.e., the known miRNA-disease associations and disease semantic similarity integrated with Gaussian interaction profile kernel similarity. As we can see the red curve in Fig. 3, FMSM manages to achieved the AUC of 0.8294 without any extra miRNA similarity, which suggests that a factored miRNA similarity based model has an ability to perform well on sparse data using a structural equation modeling approach. By introducing miRNA expression similarity, it is observed that FMSM obtains an incremental performance improvement of 7.96 and 9.54% in local LOOCV and 5-fold CV respectively. It suggests that miRNA expression similarity yielded by direct expression profiling leads to less prediction error. However, the miRNA expression similarity is still not completely covered and that we further introduced Gaussian interaction profile kernel similarity to alleviate this issue based on the known miRNA-disease associations. Accordingly, the performance of FMSM further increases 3.43 and 2.98% in local LOOCV and 5-fold CV respectively.

**Table 2:** The performance evaluation of FMSM by introducing different types of miRNA similarity in terms of 5-fold CV for 20 times

Types of miRNA similarity	The average value of AUCs
No extra miRNA similarity	0.8377+/−0.0084
MiRNA expression similarity	0.9331+/−0.0121
MiRNA similarity with expression files and Gaussian kernel	0.9629+/−0.0127

## Discussion

Several factors could be concluded as “silver bullet” solutions for the well-performing prediction of the proposed model. First, we directly extracted the miRNA expression similarity from the expression levels in 172 human tissues and cell lines. It is useful to improve the quality of miRNA similarity matrix instead of using the pairwise miRNA functional similarity inferred by Wang’s method [43]. Second, a factored miRNA similarity model is applied to learn transitive relations between miRNAs by projecting the implicit information onto two latent factor matrices. Most importantly, this model is applicable to sparse data. Third, a local scoring scheme is more suitable for top-N recommendation for each disease, rather than the global one. We have found that the known miRNA-disease associations in HMDD v2.0 are disproportional to some degree. It may bring up some misunderstanding that the diseases with less associations in HMDD v2.0 could be considered as having a low probability to potentially interact with miRNAs. It is necessary to prioritize the most potential miRNA biomarkers for various human diseases, instead of the most latent miRNA-disease associations among all unknown miRNA-disease pairs. Finally, since disease semantic similarity and miRNA expression similarity are still not completely covered, Gaussian interaction profile kernel similarity is effective to address this issue. Undoubtedly, there are some limitations inhibiting the prediction performance of FMSM. For example, it needs to take time to optimize the parameters. The proposed model cannot work on the new disease without known associated miRNAs.

## Conclusions

Increasing studies have demonstrated that miRNAs play a significant role in a wide range of biological processes, especially disease mechanisms and development. A number of miRNAs have been regarded as ideal biomarkers of disease therapy, diagnosis, prognosis and prevention. It is desirable to identify more potential miRNA biomarkers for various human diseases. However, traditional biological experiments are costly, laborious and time-consuming. Developing computational methods are anticipated to facilitate the process of miRNA biomarkers identification. In this paper, we propose a novel computational model called FMSM for inferring potential miRNA biomarkers involving in the mechanism of various disease. FMSM implicitly learns relationships between diseases and miRNAs based on a structural equation modeling approach by projecting the values in a latent space of low dimensionality. Based on the known miRNA-disease associations, miRNA expression similarity, disease semantic similarity and Gaussian interaction profile kernel similarity, all potential miRNAs are ranked prioritizing the most likely latent biomarkers for various human diseases via FMSM. The comparison experiments based on cross validation suggest that FMSM outperforms other state-of-the-art competitors and classical algorithms. In addition, the case study further demonstrates the reliable prediction of FMSM. The factored miRNA similarity based model and miRNA expression similarity has been validated to make a great contribution to an incremental performance improvement. The reliable prediction of FMSM provides an insight into the identification of potential miRNA biomarkers and aids future research efforts toward miRNA involvement in human disease mechanism.

## Methods

### MiRNA-disease association datasets

To investigate the roles of miRNAs in human disease, Li et al. [29] presented the Human MicroRNA Disease Database named HMDD v2.0, (http://www.cuilab.cn/hmdd) collecting experimentally supported miRNA and human disease associations. In this database, 5430 non-overlapping entries are provided with detailed annotations from genetics, epigenetics and circulation. These associations are involved in 383 human diseases and 495 miRNAs, whose respective cardinalities are *nd* and *nm*. In this paper, all miRNA-disease associations are represented by an adjacency matrix *U* of size *nd*×*nm*. *U* is a binary matrix, which means that if the disease *d* has been confirmed to have association with miRNA *m*, the corresponding entry in *U* denoted by *U*(*d,m*) is 1, otherwise 0. The whole set of the known miRNA-disease associations is denoted by *R*. Moreover, dbDEMC [28] and miR2Disease [27] are used as independent databases to validate the prediction lists of case studies in **Results and Discussion** section.

### MiRNA expression similarity

Betel et al. [7] proposed microRNA.org database providing miRNA expression profiles in 172 various human tissues and cell lines. Based on the hypothesis that two miRNAs tend to be closely related to the similar diseases if they have similar expression level in human tissues, all investigated miRNAs are represented by 172-dimensional vectors from the expression profiles derived from microRNA.org. To measure the miRNA expression similarity denoted as *ES*, the Person correlation coefficient was simply used as follows:

2 $$ ES\left({m}_i,{m}_j\right)=\frac{\sum \left({e}_{m_i}-\overline{e_{m_i}}\right)\left({e}_{m_j}-\overline{e_{m_j}}\right)}{\sqrt{\sum {\left({e}_{m_i}-\overline{e_{m_i}}\right)}^2\sum {\left({e}_{m_j}-\overline{e_{m_j}}\right)}^2}} $$

where *ES* is the miRNA expression similarity matrix of size *nm*×*nm*, the vectors of two miRNAs *m*_*i*_ and *m*_*j*_ are denoted as ${e}_{m_i}$ and ${e}_{m_j}$ respectively, and $\overline{e_{m_i}}$ and $\overline{e_{m_j}}$ represents the mean values of ${e}_{m_i}$ and ${e}_{m_j}$. In this way, the entity *ES(m*_*i*_*,m*_*j*_*)* is measured between 0 and 1.

### Disease semantic similarity

The National Library of Medicine (http://www.ncbi.nlm.nih.gov/) [52] provides specific MeSH descriptors to each human disease for effective classification indicating the relationship between diverse diseases. For example, the MeshID of Bacterial Infections and Mycoses is C01, while C01.252 is the counterpart of Bacterial Infections, which is categorized into a subtype of Bacterial Infections and Mycoses. In this work, we convert these relationships into respective Directed Acyclic Graphs (DAGs) to measure the similarity between any two diseases. Given a disease *D*, its DAG can be represented as *DAG*(*D*) = (*T*(*D*), *E*(*D*)), where *T(D)* is a node set of *D* and its ancestor nodes while *E(D)* refers to the edge set of all direct edges from parent nodes to child nodes. In this way, we assume that disease *D* locates in the root layer, so the contribution score for the semantic value of disease *D* itself is set to 1. Empirically, the contribution of any *D*’s ancestor disease *d* in *DAG(D)* to the semantic value of *D* could be inversely decreased, as the path elongates from *D* to *d*. Based on *DAG(D)*, such kind of numerical calculation can be formulated as follows:

3 $$ \left\{\begin{array}{l}{C}_D(d)=1\kern15.5em if\ d=D\\ {}{C}_D(d)=\max \left\{{\varDelta}_{\ast }{C}_D\left({d}^{\hbox{'}}\right)|{d}^{\hbox{'}}\in children\ of\ d\right\}\kern1.5em if\ d\ne D\end{array}\right. $$

where △ is a contribution decay parameter in the range from 0 to 1. In this paper, △ is set to 0.5 according to the previous work [38, 53]. We defined *AC(D)* as the aggregate semantic value of disease *D* for further illustration, i.e. $\mathrm{AC}\left(\mathrm{D}\right)={\sum}_{d\in T(D)}{C}_D(d)$. It is obvious that if any two diseases share larger common parts of their DAGs, the semantic similarity score between themselves should be assigned a greater weight. Based on this assumption, the disease semantic similarity matrix of size *nd*×*nd* could be calculated as:

4 $$ SS\left({d}_i,{d}_j\right)=\frac{\sum_{t\in T\left({d}_i\right)\cap T\left({d}_j\right)}\left({C}_{d_i}(t)+{C}_{d_j}(t)\right)}{AC\left({d}_i\right)+ AC\left({d}_j\right)} $$

### Gaussian interaction profile kernel similarity

To alleviate the data sparsity problem of similarity matrix, Gaussian interaction profile kernel similarity for both miRNA and disease are calculated based on the hypothesis [43, 54, 55] that any two miRNAs/diseases have a greater opportunity to be potentially related if they share more common diseases/miRNAs respectively. It motivates us to introduce Gaussian interaction profile kernel for the inference of miRNA- and disease- similarity by exploiting the implicit topologic information of the miRNA-disease association matrix, i.e. matrix *U*. The process of the inferred disease similarity could be roughly divided into two steps: (1) given any two diseases *d*_*i*_ and *d*_*j*_, their interaction profiles are denoted as two binary vectors *IP(d*_*i*_*)* and *IP(d*_*j*_*)* respectively. They represent the set of associations between *di*/*dj* and each miRNA, i.e. the *ith* and *jth* column of matrix *U*. Then, Gaussian interaction profile kernel similarity matrix *KD* of size *nd*×*nd* could be defined as follows:

5 $$ KD\left({d}_i,{d}_j\right)=\exp \left(-{\gamma}_d{\left\Vert IP\left({d}_i\right)- IP\left({d}_j\right)\right\Vert}^2\right) $$

where parameter *γ*_*d*_ controls the kernel bandwidth. (2) *γ*_*d*_ needs to be updated by normalizing a new bandwidth parameter *γ*′_*d*_ divided by the average value of associated miRNAs for each disease.

6 $$ {\gamma}_d={\gamma^{\hbox{'}}}_d/\left(\frac{1}{nd}\sum \limits_{i=1}^{nd}{\left\Vert IP\left({d}_i\right)\right\Vert}^2\right) $$

Here, *γ*′_*d*_ is set to 1 for simplifying the calculation based on previous research [56], rather than following the original method [57].

For miRNAs, Gaussian interaction profile kernel similarity *KM* of size *nm*×*nm* could be calculated in the similar way as

7 $$ KM\left({m}_i,{m}_j\right)=\exp \left(-{\gamma}_m{\left\Vert IP\left({m}_i\right)- IP\left({m}_j\right)\right\Vert}^2\right) $$

8 $$ {\gamma}_m={\gamma^{\hbox{'}}}_m/\left(\frac{1}{nm}\sum \limits_{i=1}^{nm}{\left\Vert IP\left({m}_i\right)\right\Vert}^2\right) $$

where *γ*′_*m*_ is also set to 1. It is worthwhile to note that *KD* and *KM* should be recalculated when implementing each cross validation.

### Integrated similarity matrices for miRNA and disease

MiRNA expression similarity *ES* and disease semantic similarity *SS* are effective to construct the respective similarity matrices for miRNA and disease. However, neither *ES* or *SS* cover all investigated miRNAs and diseases. Accordingly, we utilized Gaussian interaction profile kernel similarity for those uncovered miRNAs and diseases (i.e. *KM* and *KD*) to fill in the missing values in *ES* and *SS*. Therefore, the integrated similarity matrices for miRNA and disease (*S*_*m*_ and *S*_*d*_) can be defined as follows:

9 $$ {S}_m\left({m}_i,{m}_j\right)=\frac{ES\left({m}_i,{m}_j\right)+ KM\left({m}_i,{m}_j\right)\ }{2} $$

10 $$ {S}_d\left({d}_i,{d}_j\right)=\left\{\begin{array}{l} SS\left({d}_i,{d}_j\right)\kern1.75em {d}_i\ \mathrm{and}\ {d}_j\ \mathrm{has}\ \mathrm{semantic}\ \mathrm{similarity}\\ {} KD\left({d}_i,{d}_j\right)\kern8.25em \mathrm{otherwise}\end{array}\right. $$

### FMSM

**Fig. 4:**
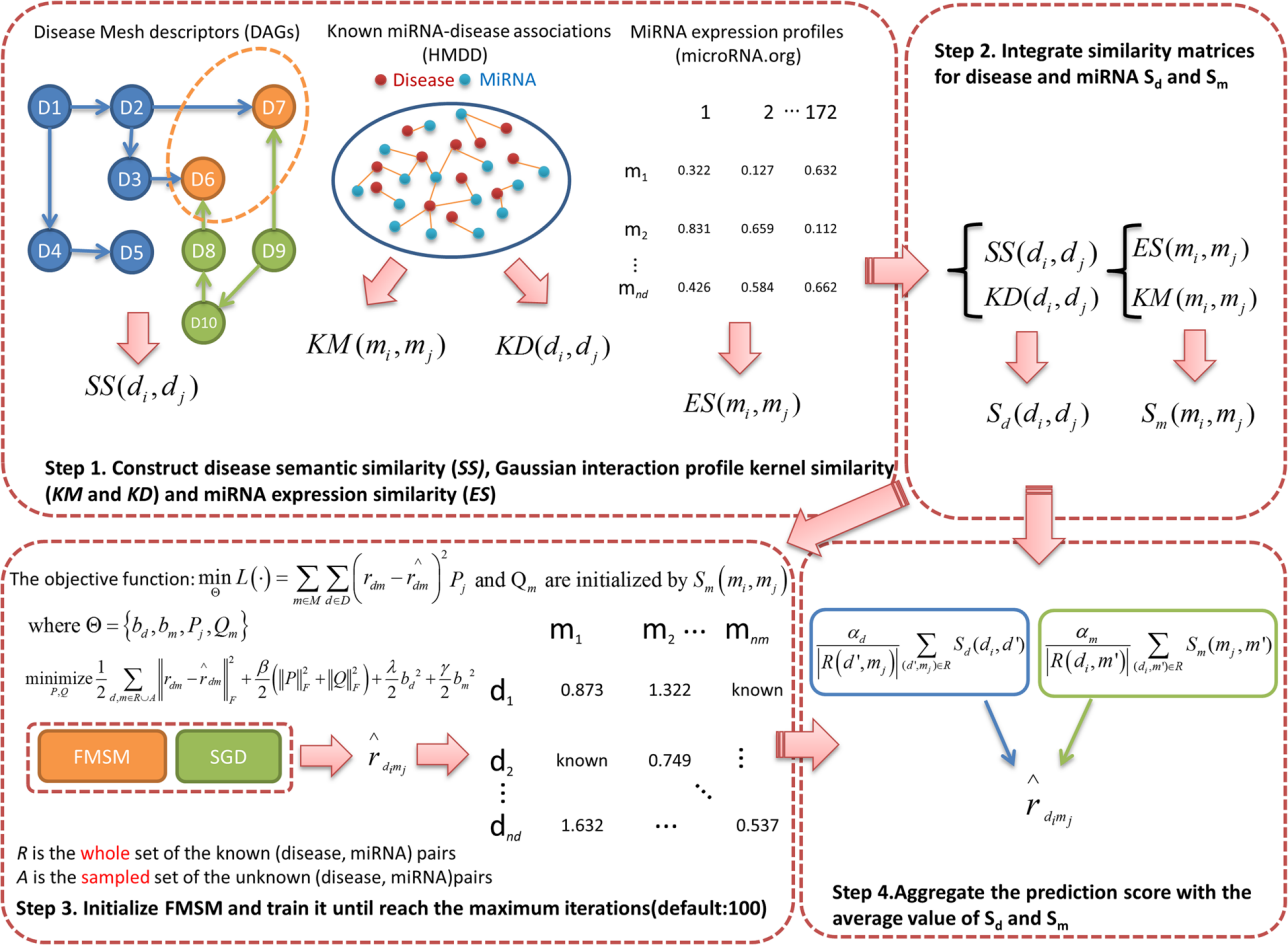
Flowchart of FMSM. Based on the known miRNA-disease associations, miRNA expression similarity, disease semantic similarity and Gaussian interaction profile kernel similarity, the latent miRNA biomarkers for various diseases were prioritized based on the prediction score (${r}_{d_i{m}_j}^{\hat{\mkern6mu} }$)

Inspired by the idea of FISM [37] in user-item recommender problem, we developed a novel Factored MiRNA Similarity Model (FMSM) to predict miRNA molecules involving in the mechanism of various diseases. FMSM learns the miRNA-miRNA similarity matrix as a product of two latent factor matrices. The flowchart of FMSM is shown in Fig. 4. To allow readers more easily to follow the model description, the parameter settings are tabulated in Table 3. Using a structural equation modeling approach leads to better estimators for generating high quality prediction results even on sparse datasets (sparsity = 2.86%, 5430/nm/nd*100%).

**Table 3:** The parameter settings of FMSM

Parameter	Setting
Size of the known miRNA-disease associations (R)	5430
Number of diseases (nd)	383
Number of miRNA (nm)	495
Regularization weights for latent factor matrices *P* and Q (β, λ and γ)	Β = λ = γ = 0.1
Maximum number of iterations (T)	100
Regulation weights for average values of *R*(*d*_*i*_, *m*^′^) and *R*(*d*^′^, *m*_*j*_) (W_d_ and W_m_)	1
Learning rate (η)	0.01
Sample factor (ƿ)	3

Based on the known miRNA-disease association network, we calculate the loss to measure the difference between the truth value *r*_*dm*_ and the estimated value ${r}_{dm}^{\hat{\mkern6mu} }$ by using the squared error loss function as follows:

11 $$ L\left(\cdot \right)=\sum \limits_{m\in M}\sum \limits_{d\in D}{\left({r}_{dm}-\overset{\wedge }{r_{dm}}\right)}^2 $$

where *D* and *M* denote the sets of diseases and miRNAs, respectively. *r*_*dm*_ is the truth value, namely if disease *d* has been confirmed to have association with miRNA *m*, *r*_*dm*_=1 otherwise 0. ${r}_{dm}^{\hat{\mkern6mu} }$, the estimated value, could be calculated as

12 $$ {r}_{dm}^{\wedge }={b}_d+{b}_m+\frac{1}{{\left({n}_d^{+}-1\right)}^{\alpha }}\sum \limits_{j\in {R}_d^{+}\backslash \left\{m\right\}}{p}_j{q_m}^T $$

where *b*_*d*_ and *b*_*m*_ are floating points representing the biases of disease and miRNA, respectively. ${n}_d^{+}$ is the number of miRNAs associated with disease *d*. α is a disease specified factor between 0 and 1. ${R}_d^{+}\backslash \left\{m\right\}$ represents the set of miRNAs associated with disease *d* excluding the miRNA *m*, whose value is being estimated. It is important to do this exclusion for conforming to regression model according to the structural equation modeling. *p*_*j*_ and *q*_*m*_ are two learned miRNA latent factors from matrices *P* and *Q*, respectively.

*P* and *Q* are two matrices of size *nm*×*d* (where *d* < nm) and are originally initialized by miRNA similarity *S*_*m*_. Since FISM was proposed for the user-item recommender problem involving three large datasets (sizes of 943*1178, 6079* 5641 and 7558*3951 respectively). Considering its practical application prospect, its authors attempted to make a tradeoff between time consumption and accuracy. For a fast recommendation, they set *P* and *Q* as two low dimensional latent factor matrices. However, in this work, time consumption is no longer important. The dimensions of *P* and *Q* can be higher for better estimating the similarity. And based on 5-fold CV, FMSM with high dimensions of *P* and *Q* achieved higher AUC value by around 2.6% than low randomized dimensions’ did. Obviously, if we minimize the squared error loss function L(∙), Eqs (11) and (12) can be converted into Eq. (13) by minimizing the following regularized optimization problem:

13 $$ \underset{P,Q}{\operatorname{minimize}}\frac{1}{2}\sum \limits_{d,m\in R\cup A}{\left\Vert {r}_{dm}-{\overset{\wedge }{r}}_{dm}\right\Vert}_F^2+\frac{\beta }{2}\left({\left\Vert P\right\Vert}_F^2+{\left\Vert Q\right\Vert}_F^2\right)+\frac{\lambda }{2}{b_d}^2+\frac{\gamma }{2}{b_m}^2 $$

where β, λ and γ are the regularization weights for latent factor matrices *P* and *Q*, disease bias *b*_*d*_ and miRNA bias *b*_*m*_ respectively (β = λ = γ∈{0.001, 0.01, 0.1}, we use 0.1 in this work).

All entries of training set include *R* and the sampled set of unknown miRNA-disease associations *A*. It helps reduce the computational complexity for optimization. To solve the optimization problem of Eq. (13), we exploit a Stochastic Gradient Descent (SGD) algorithm, whose detailed pseudo-code is provided in Algorithm 1. The training process is repeated until the maximum number of iterations has reached a predefined threshold (default: 100). In this way, the estimated score of each unknown pair in *U* can be computed, i.e. ${r}_{dm}^{\hat{\mkern6mu} }$. Finally, we need to aggregate ${r}_{dm}^{\hat{\mkern6mu} }$ with the integrated similarity matrices for disease and miRNA, i.e. *S*_*d*_ and *S*_*m*_. Given an unknown miRNA-disease association in *U*, e.g. *U*(*d*_*i*_*,m*_*j*_), a set of miRNAs associated with *d*_*i*_ and a set of diseases associated with *m*_*j*_ are denoted by *R*(*d*_*i*_, *m*^′^) and *R*(*d*^′^, *m*_*j*_), respectively. Empirically, we add the average values of *R*(*d*_*i*_, *m*^′^) and *R*(*d*^′^, *m*_*j*_) to ${r}_{d_i{m}_j}^{\hat{\mkern6mu} }$ with regulation weights *W*_*d*_ and *W*_*m*_, which could be defined as follows:

14 $$ {r}_{d_i{m}_j}^{\wedge }={r}_{d_i{m}_j}^{\wedge }+\frac{W_d}{\left|R\left({d}^{\hbox{'}},{m}_j\right)\right|}\sum \limits_{\left({d}^{\hbox{'}},{m}_j\right)\in R}{S}_d\left({d}_i,{d}^{\hbox{'}}\right)+\frac{W_m}{\left|R\left({d}_i,{m}^{\hbox{'}}\right)\right|}\sum \limits_{\left({d}_i,{m}^{\hbox{'}}\right)\in R}{S}_m\left({m}_j,{m}^{\hbox{'}}\right) $$

where *W*_*d*_ = *W*_*m*_ = 1. ${r}_{d_i{m}_j}^{\hat{\mkern6mu} }$ represents the predicted score for the potential association between *d*_*i*_ and *m*_*j*_. Namely, the higher value of ${r}_{d_i{m}_j}^{\hat{\mkern6mu} }$ they obtain, the more likely they are related.

The FMSM algorithm can be summarized as following steps:

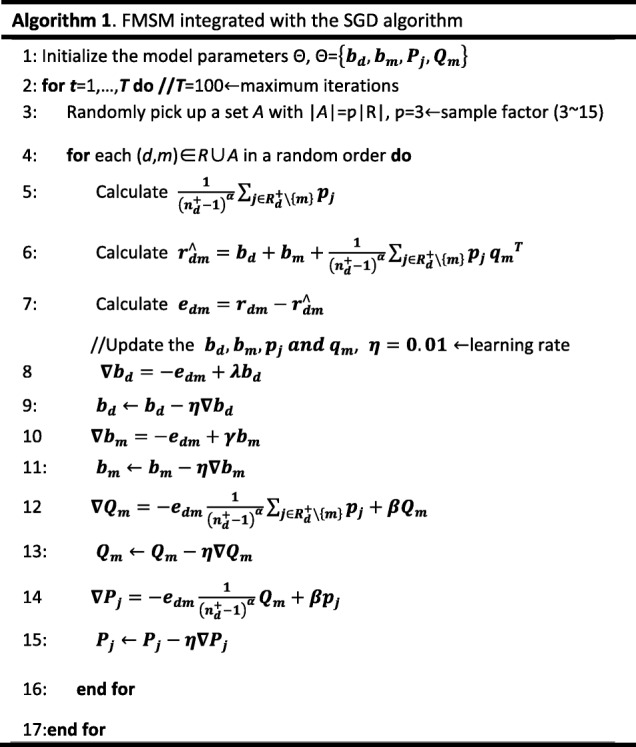



## Additional file

Additional file 1: The prediction list of most latent miRNA biomarkers for various investigated diseases has been publicly released. (XLSX 3187 kb)

## Abbreviations

AUC: Area under ROC curve
BCR: B-cell receptor
CN: Colonic Neoplasms
CV: 5-fold cross validation
DAGs: Directed Acyclic Graphs
FMSM: Factored MiRNA Similarity Model
FPR: False positive rate
LOOCV: Leave-one-out cross validation 5-fold
miRNA: MicroRNA
MTDN: MiRNA target-dysregulated network
MTIs: MiRNA-target interactions
PPI: Protein-protein interaction
RLS: Regularized Least Squares
ROC: The receiver operating characteristic
TPR: True positive rate
